# Extracellular Vesicles Derived from Allergen Immunotherapy-Treated Mice Suppressed IL-5 Production from Group 2 Innate Lymphoid Cells

**DOI:** 10.3390/pathogens11111373

**Published:** 2022-11-17

**Authors:** Masaya Matsuda, Seito Shimizu, Kazuyuki Kitatani, Takeshi Nabe

**Affiliations:** Laboratory of Immunopharmacology, Faculty of Pharmaceutical Sciences, Setsunan University, 45-1 Nagaotoge cho, Hirakata 573-0101, Japan

**Keywords:** airway hyperresponsiveness, allergy, asthma, extracellular vesicle, group 2 innate lymphoid cell, interleukin-5, subcutaneous immunotherapy

## Abstract

Allergen immunotherapy (AIT), such as subcutaneous immunotherapy (SCIT), is a treatment targeting the causes of allergic diseases. The roles of extracellular vesicles (EVs), bilayer lipid membrane blebs released from all types of cells, in AIT have not been clarified. To examine the roles of EVs in SCIT, it was analyzed whether (1) EVs are phenotypically changed by treatment with SCIT, and (2) EVs derived from SCIT treatment suppress the function of group 2 innate lymphoid cells (ILC2s), which are major cells contributing to type 2 allergic inflammation. As a result, (1) expression of CD9, a canonical EV marker, was highly up-regulated by SCIT in a murine model of asthma; and (2) IL-5 production from ILC2s in vitro was significantly decreased by the addition of serum EVs derived from SCIT-treated but not non-SCIT-treated mice. In conclusion, it was indicated that EVs were transformed by SCIT, changing to a suppressive phenotype of type 2 allergic inflammation.

## 1. Introduction

The number of patients with allergies such as rhinitis has gradually increased worldwide [1]. Treatment for allergic diseases utilizes two well-accepted approaches; conventional pharmacotherapy and allergen-specific immunotherapy (AIT). In conventional pharmacotherapy, anti-leukotrienes [2], anti-histamines [3], inhaled and systemic corticosteroids [4], and monoclonal antibodies against type 2 cytokines [5,6] can control allergic symptoms. However, it is well-known that allergic symptoms relapse after cessation of pharmacotherapy. Therefore, the development of treatments against the causes of allergic diseases is required.

AIT has been recognized as the only treatment that can modify the natural course of allergy by inducing immune tolerance to allergens [7]. Since the initial publication by Noon et al. [8], the clinical effectiveness of AIT against allergic diseases has been demonstrated in numerous studies [9,10,11,12,13]. AIT is mainly conducted in two forms: subcutaneous immunotherapy (SCIT) and sublingual immunotherapy [14]. However, AIT remains underused because: (1) long-term treatment is required to acquire sustainable remission of allergic symptoms [15,16], (2) some patients are non-responders [17,18], and (3) some patients have rare anaphylactic reactions [19,20]. Therefore, a deeper understanding of the mechanisms is required to develop more-effective and safer AIT.

It has been generally recognized that type 2 allergic inflammation is orchestrated by type 2 helper T (Th2) cells and group 2 innate lymphoid cells (ILC2s), both of which produce type 2 cytokines such as interleukin (IL)-5, IL-13, and IL-4 [21]. Recent research regarding type 2 allergic inflammation has shed light on ILC2s, because ILC2s produce a large amount of IL-5 and IL-13 in response to epithelial-derived cytokines, IL-33, and thymic stromal lymphopoietin (TSLP) [22,23]. Our previous study using a murine model of asthma demonstrated that ILC2s produced more IL-5 in vitro than CD4^+^ T cells, mainly consisting of Th2 cells [24]. The produced IL-5 and IL-13 from ILC2s induce eosinophilic infiltration into allergic inflamed tissues and the development of airway hyperresponsiveness (AHR) [22,23]. Thus, ILC2 regulation is crucial for alleviating allergic symptoms. In recent years, several groups [25,26] reported that AIT down-regulated IL-5 and IL-13 production from ILC2s and suppressed the proliferation of ILC2s in mice and humans. However, the mechanisms underlying the effects of AIT on the functions of ILC2s remain to be clarified.

Extracellular vesicles (EVs), bilayer lipid membrane blebs with a diameter of approximately 30–100 nm, are released from all types of cells [27]. EVs contain host cell-derived proteins, such as tetraspanin proteins CD9 and CD63, adhesion molecules, and immune regulator molecules [28,29]. Tetraspanin proteins such as CD9 and CD63 have usually been recognized as EV membrane markers, because they have crucial roles in EV-associated events such as adhesion, invasion, and membrane fusion to recipient cells [30,31]. Besides proteins, EVs are abundantly loaded with host cell-derived RNAs, such as messenger RNAs and microRNAs (miRNAs) [32]. The tetraspanin proteins work when EVs are captured by recipient cells, inducing functional changes in recipient cells. It has been reported that EVs play roles in the pathogenesis of allergies: Paredes et al. [33] reported that EVs in bronchoalveolar lavage fluid (BALF) derived from patients with mild allergic asthma to birch pollen promoted leukotriene C_4_ and IL-8 releases in human bronchial epithelial cells. Canas et al. [34] also demonstrated that eosinophil-derived EVs from asthmatic patients contributed to airway remodeling by inducing proliferation of airway muscle cells. Taken together, AIT induced dynamic immunological changes (reviewed in [35,36]), such as decreases in inflammatory cells and induction of anti-inflammatory cells, whereas the roles of EVs in AIT remain to be clarified.

We previously established an ovalbumin (OVA)-induced asthmatic model of mice [24,37], in which SCIT exerted significant suppression of development of AHR and airway remodeling. Moreover, SCIT treatment significantly suppressed ILC2 proliferation in the lung (unpublished data). Therefore, this murine model can be utilized for analyzing the mechanisms of SCIT. In this study, in order to clarify the roles of EVs in AIT using the murine model, (1) SCIT-induced phenotype changes of EVs and (2) suppressive effects of the EVs on IL-5 production from ILC2s were analyzed.

**Figure 1:**
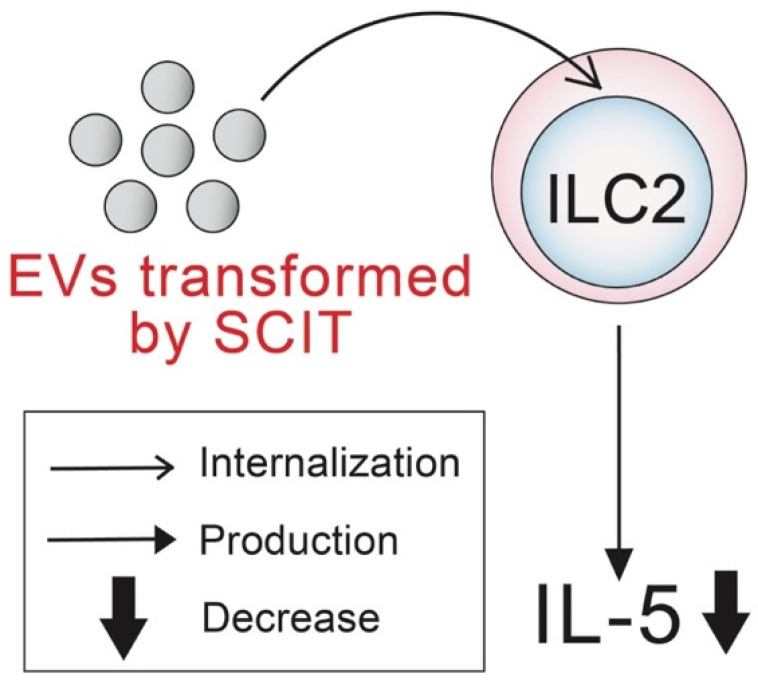
Graphical abstract of this study. Extracellular vesicles (EVs) in the bloodstream were transformed by subcutaneous immunotherapy (SCIT), acquiring ability to inhibit interleukin 5 (IL-5) production from group 2 innate lymphoid cells (ILC2s).

The graphical abstract of this study is described in Figure 1.

## 2. Results

### 2.1. Effects of SCIT on the Development of AHR and IL-5 Production in BALF

**Figure 2:**
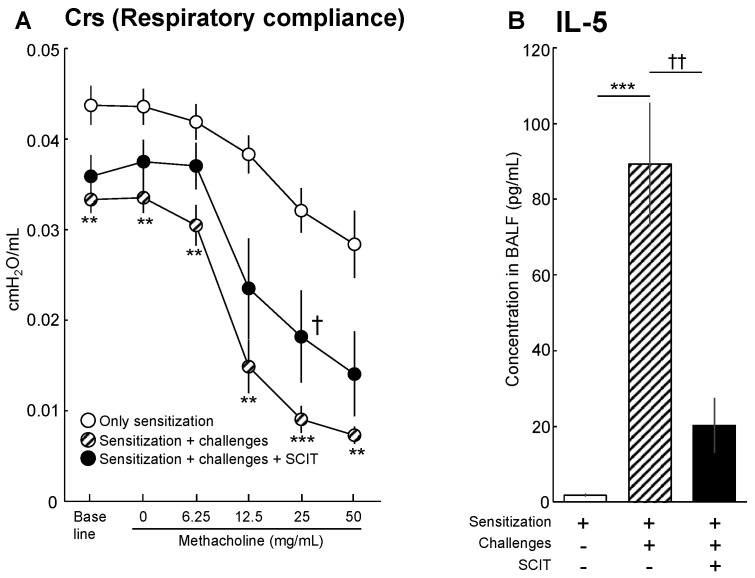
Effects of subcutaneous immunotherapy (SCIT) on airway hyperresponsiveness (**A**) and an amount of interleukin 5 (IL-5) in bronchoalveolar lavage fluid (BALF) (**B**) of ovalbumin (OVA)-induced asthmatic mice. BALB/c mice were sensitized with intraperitoneal (i.p.) OVA + Al(OH)_3_ on days 0 and 14, followed by intratracheal OVA challenges on days 35, 36, 37, and 40 at a dose of 5 μg/animal. SCIT treatment was applied by subcutaneous injection of OVA (1 mg/animal) on days 21, 23, and 25. Twenty-four hours after the 4th OVA challenge (day 41), the development of airway hyperresponsiveness (AHR) was analyzed. Respiratory compliance (Crs) is shown as the maximum value after each methacholine challenge. Following analysis of AHR, bronchoalveolar lavage was conducted. IL-5 concentration in BALF was quantified by ELISA. Each point and column represent the mean ± SEM of 4 or 5 animals. **: *p* < 0.01 and ***: *p* < 0.001 versus only sensitization. †: *p* < 0.05 and ††: *p* < 0.01 versus sensitization + challenges.

To clarify the effects of SCIT on respiratory function, AHR to methacholine was measured by the forced oscillation technique using FlexiVent. AHR to methacholine was developed in OVA-challenged asthmatic mice when respiratory compliance (Crs), which reflects the flexibility of the lung, was assessed as a parameter of airway function (Figure 2A). As shown in Figure 2A, the Crs of OVA-challenged mice declined even in response to lower concentrations of methacholine than “only sensitization” mice, and the magnitude of the decline at relatively high concentrations of methacholine was considerable compared with that of the “only sensitization” group. On the other hand, the Crs decline was significantly improved by treatment with SCIT.

Figure 2B represents the effect of SCIT on IL-5 production in the lung of OVA-challenged asthmatic mice. The amount of IL-5 in BALF was significantly increased in OVA-challenged asthmatic mice. Augmented IL-5 production was markedly decreased in SCIT-treated asthmatic mice (Figure 2B).

### 2.2. Phenotypes of EVs Derived from Sera of Non-SCIT-Treated and SCIT-Treated Asthmatic Mice

**Figure 3:**
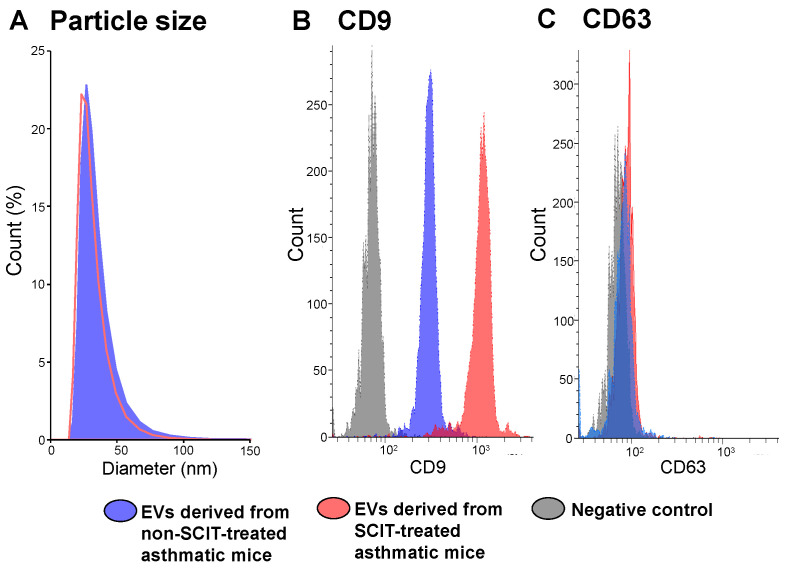
Particle sizes (**A**), and expression levels of CD9 (**B**) and CD63 (**C**) of extracellular vesicles (EVs) derived from the non-subcutaneous immunotherapy (SCIT)-treated and SCIT-treated asthmatic mice. Twenty-four hours after the 4th challenge, sera were collected. EVs were obtained from sera by ExoQuick. The particle sizes of EVs were detected using dynamic light scattering. The expression levels of CD9 and CD63 were analyzed by flow cytometry.

Figure 3 represents particle sizes and CD9 and CD63 expression on EVs derived from sera of non-SCIT-treated and SCIT-treated asthmatic mice. Particle sizes of EVs did not differ between the two groups (Figure 3A). On the other hand, the expression level of CD9 on EVs derived from SCIT-treated asthmatic mice was markedly higher than that from non-SCIT-treated asthmatic mice (Figure 3B). No difference in CD63 expression level was observed (Figure 3C).

### 2.3. Effects of EVs on IL-5 Production from ILC2s

**Figure 4:**
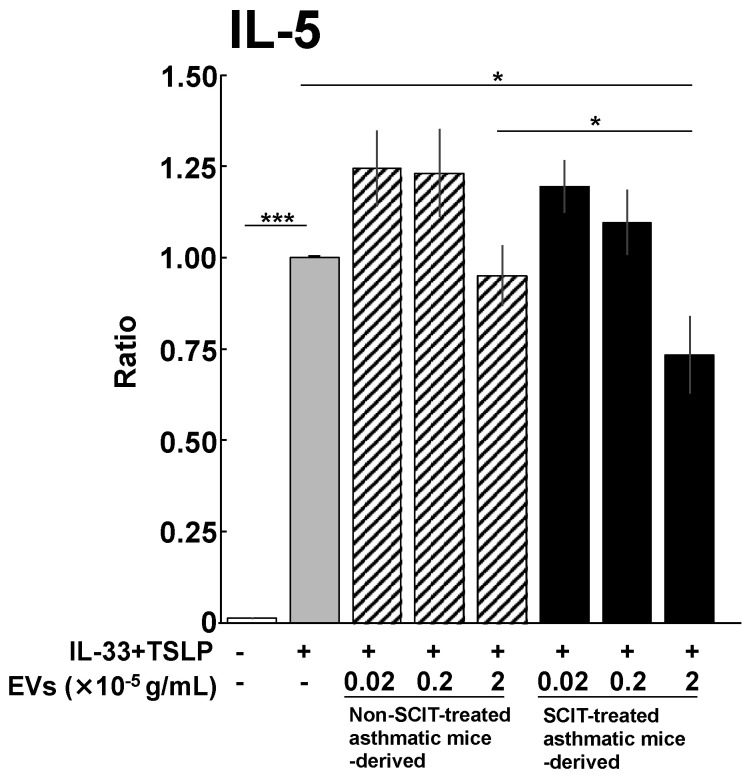
Effects of extracellular vesicles (EVs) derived from sera of the non-subcutaneous immunotherapy (SCIT)-treated or SCIT-treated asthmatic mice on interleukin 5 (IL-5) production from group 2 innate lymphoid cells (ILC2s). All lung lobes were collected 24 h after the 4th ovalbumin (OVA) challenge. ILC2s in the dispersed lung tissue were detected and sorted as lineage^−^ CD45^+^ CD278^+^ CD90.2^+^ ST2^+^ cells by flow cytometry. The sorted ILC2s were cultured for 72 h in the presence of IL-33 and TSLP with or without EVs. After the culture, cell culture supernatants were collected, followed by measurement of IL-5 by ELISA. Each column represents the mean ± SEM of ratio to the control (IL-33/TSLP—stimulated, without EVs) of 5 experiments. *: *p* < 0.05 and ***: *p* < 0.001.

As shown in Figure 4, lung ILC2s derived from non-SCIT-treated asthmatic mice abundantly produced IL-5 in response to combined stimulation with IL-33 and TSLP in vitro. IL-5 production from ILC2s was not suppressed by EVs derived from non-SCIT-treated asthmatic mice. On the other hand, EVs derived from SCIT-treated asthmatic mice significantly ameliorated IL-5 production from ILC2s. 

## 3. Discussion

Although there has been accumulating evidence that EVs are involved in the pathogenesis of allergic diseases, the roles of EVs in AIT have not been elucidated. In this study, we hypothesized that the phenotype of EVs was transformed by treatment with SCIT, and that transformed EVs suppress the functions of effector cells, leading to amelioration of type 2 allergic inflammation. We demonstrated that the development of AHR and IL-5 production in the lungs were significantly suppressed by SCIT in vivo (Figure 2). Moreover, EVs derived from serum of SCIT-treated asthmatic mice significantly suppressed IL-5 production from lung ILC2s (Figure 4). To the best of our knowledge, this is the first report to demonstrate that EVs are involved in the suppression of effector cells in the mechanisms of AIT.

The expression level of CD9, but not CD63, on serum EVs was dramatically up-regulated by SCIT. CD9 is a canonical EV marker, which participates in the events when EVs are captured and incorporated into recipient cells [38,39]. CD9 is also involved in the enhancement and maintenance of IL-10 secretion in murine and human antigen-presenting cells [40,41]. Indeed, Suzuki et al. [42] demonstrated that CD9 suppressed lipopolysaccharide-induced lung inflammation by inducing IL-10-producing macrophages in mice. IL-10 suppresses type 2 cytokine production and the proliferation of ILC2s, which constantly express IL-10 receptor α subunit [43,44,45]. Therefore, the increase in CD9 on the EVs may suppress the activation of ILC2s through the induction of IL-10-producing macrophages.

Although the source of CD9-highly-expressing EVs is unclear, one possible source is regulatory B (Breg) cells. Several reports [46,47] demonstrated that the number of Breg cells was significantly increased in the peripheral blood of patients with allergic diseases by SCIT. CD9 is highly expressed in Breg cells in mice and humans [48,49,50]. It has been reported that highly expressed proteins in host cells tended to be preferentially loaded into EVs [51,52,53]. Therefore, SCIT-induced Breg cells may produce CD9-highly-expressing EVs. Moreover, Kang et al. [54] demonstrated that Breg cell-derived EVs suppressed neuroinflammation and autoimmune uveitis by inducing IL-10- and IL-35-secreting regulatory T (Treg) cells in mice. Therefore, the induction of Breg cells by SCIT may be associated with increases in the expression of CD9 on EVs.

SCIT induces not only Breg cells but also Treg cells [55,56]. Our group previously demonstrated that Treg cells were significantly increased in the lungs of an SCIT-treated asthmatic murine model [37], and peripheral blood of patients with Japanese cedar pollinosis [46]. Treg cells can also release EVs, which are captured by effector T cells [57,58] and dendritic cells [59], followed by suppression of activation. We also demonstrated that IL-5 production from murine ILC2s stimulated with IL-33 and TSLP was significantly down-regulated in the presence of Treg-derived EVs in a concentration-dependent manner (unpublished data). Treg cell-derived EVs expressed an ectoenzyme CD73 in their extracellular membrane [51], and CD73 converted adenosine triphosphate into adenosine in inflammatory conditions [60]. Two groups [61,62] reported that adenosine contributed to the suppression of IL-5 and IL-13 production from ILC2s via binding to adenosine A_2A_ or A_2B_ receptors. Therefore, EVs derived from SCIT-induced Treg cells may also exert suppression of IL-5 and IL-13 production from lung ILC2s.

Not only ILC2 but also Th2 cells produce IL-5, leading to the development of AHR [21]. Our group recently clarified that the numbers of both ILC2 and Th2 cells in the lungs of OVA-induced asthmatic mice were significantly decreased by SCIT (unpublished data). Therefore, SCIT could alleviate the development of AHR via decreasing the numbers of ILC2 and Th2 cells in lungs.

IL-5 production from ILC2 tended to be increased in the presence of the low-doses of EVs regardless of SCIT, and inversely decreased in the presence of the high-dose of EVs derived from SCIT-treated asthmatic mice. CD9 on EV surfaces is a pleiotropic molecule that is associated with not only immune regulation [40,41,42] but also EV adhesion and invasion to recipient cells [30,31]. Although the EV concentration in serum was unclear, SCIT may have raised EV level in the circulation to a certain concentration, at which the EVs down-regulated ILC2 functions.

EVs contain not only proteins but also miRNAs [32]. miRNAs interfere with the transcription of various genes in recipient cells, followed by transformation of the functions. In recent years, it has been reported that the miRNA expression patterns in blood and sputum were dramatically changed in allergic patients after AIT, including SCIT and insect venom immunotherapy (VIT) [63,64,65,66]. Specjalski et al. [65] demonstrated that the expression levels of 11 miRNAs, including let-7d and miR-143, were significantly up-regulated during wasp VIT. Let-7d affected human T cells to inhibit the expression of IL-13 [67], which has been known to contribute to the development of AHR [68]. miR-143 was involved in down-regulation of IL-13 receptor alpha 1 subunit expression on human mast cells, followed by inhibition of the cell proliferation [69,70]. Taken together, the expression levels of miRNAs, which interfere with the IL-13 signaling, may be up-regulated on serum EVs by SCIT.

In conclusion, SCIT may induce not only immunological changes but also phenotype changes in EVs. The transformed EVs suppressed the development of AHR by inhibiting ILC2 activation. In recent years, Boonpiyathad et al. [71] reported that the number of ILC2s was not decreased in the peripheral blood of non-responders to AIT. Transformed EVs by SCIT are expected to be a useful therapeutic tool. In addition, unlike the usage of allergen extracts, EVs do not theoretically induce anaphylactic reactions. Further understanding of suppressive mechanisms by transformed EVs may lead to the development of more-effective and safer AIT.

## 4. Materials and Methods

### 4.1. Sensitization, SCIT, and Challenges

**Figure 5:**
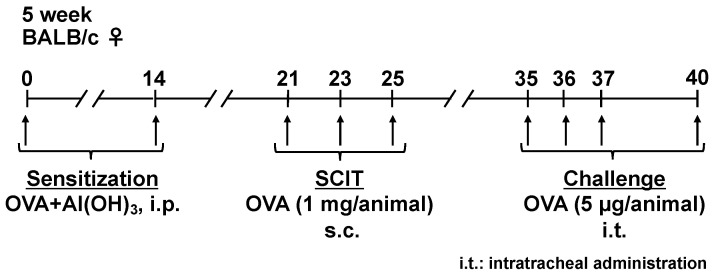
Schedule for sensitization, subcutaneous immunotherapy (SCIT), and challenges. Mice were sensitized by intraperitoneal (i.p.) injection with ovalbumin (OVA) + Al(OH)_3_ on days 0 and 14. Sensitized mice were treated with subcutaneous (s.c.) injection of OVA at a dose of 1 mg/animal on days 21, 23, and 25. The mice were challenged by intratracheal (i.t.) administration of OVA at a dose of 5 μg/animal on days 35, 36, 37, and 40 under inhalation anesthesia with isoflurane.

Sensitization, SCIT, and challenges were conducted in accordance with our previous report [37], as follows (Figure 5). Briefly, 5-week-old BALB/c mice (Japan SLC, Hamamatsu, Shizuoka, Japan) were sensitized by i.p. injections with OVA (Grade V; Sigma-Aldrich, St. Louis, MO, USA) adsorbed to Al(OH)_3_ at a dose of 50 μg OVA/2 mg Al(OH)_3_/0.5 mL saline/animal on days 0 and 14. Seven days after the second sensitization, 0.5% OVA solution was injected subcutaneously at 200 μL (1 mg OVA)/animal on days 21, 23, and 25. The mice were intratracheally challenged with 0.02% OVA solution at 25 μL (5 μg OVA) on days 35, 36, 37, and 40 under inhalation anesthesia with isoflurane (Fujifilm, Osaka, Japan). 

### 4.2. Measurement of AHR

Measurement of AHR was conducted as reported previously [72]. Briefly, AHR to methacholine was measured using the forced oscillation technique with a Flexivent FV-FX1 (SCIREQ, Montreal, QC, Canada). At 24 h after the 4th challenge, the recipient mice were anesthetized with pentobarbital (70 mg/kg) and xylazine (12 mg/kg), followed by methacholine (acetyl-β-methylcholine chloride, Sigma-Aldrich, St. Louis, MO, USA) challenges at concentrations ranging from 0 to 50 mg/mL (0, 6.25, 12.5, 25, and 50 mg/mL) for 12 s each. After each methacholine challenge, Crs, which represents respiratory flexibility, was measured using eight repeats. Crs is shown as a maximal value after each methacholine challenge.

### 4.3. Quantitative Analysis of IL-5 in BALF

After the measurement of AHR, bronchoalveolar lavage was conducted in the right lung lobes, as reported previously [73,74]. The obtained BALF was centrifuged, followed by collection of the supernatant. IL-5 concentration in BALF was determined using an IL-5 mouse ELISA kit (Thermo Fisher Scientific, Waltham, MA, USA).

### 4.4. Isolation of EVs

Twenty-four hours after the 4th challenge, non-SCIT-treated and SCIT-treated asthmatic mice were anesthetized with pentobarbital and xylazine as described above, followed by collection of whole blood from the abdominal vena cava. The whole blood was incubated in a water bath at 25 °C for 30 min. Then, the blood was incubated in a refrigerator at 4 °C overnight. After the incubation, the blood samples were centrifuged at 1200× *g* for 30 min at 4 °C, followed by serum collection.

In accordance with the manufacturer’s protocol, EVs were isolated from serum using total exosome isolation reagent (from serum) (Thermo Fisher Scientific, Waltham, MA, USA). Briefly, the serum samples were centrifuged at 2000× *g* for 30 min to remove cells and debris. The supernatants were transferred into new tubes, followed by adding 0.2 volumes of the total exosome isolation (from serum) reagent. The samples were incubated at 4 °C for 30 min, then centrifuged at 10,000× *g* for 30 min at room temperature. The obtained pellets were suspended with phosphate-buffered saline (PBS).

The concentration of EVs was determined using a BCA protein assay kit (Thermo Fisher Scientific, Waltham, MA, USA).

### 4.5. Analyses of CD9 and CD63 Expression Levels and Particle Sizes of EVs

In accordance with the manufacturer’s instructions, the expression levels of EV markers CD9 and CD63 were analyzed using a PS capture exosome flow cytometry kit (Fujifilm, Tokyo, Japan). Briefly, exosome capture beads (Fujifilm, Tokyo, Japan) and exosome binding enhancer (Fujifilm, Tokyo, Japan) were added to the EV samples, followed by incubation at room temperature for 1 h. After washing with exosome binding enhancer-containing washing buffer (Fujifilm, Tokyo, Japan), the bead-bound EVs were stained with allophycocyanin (APC)-conjugated anti-mouse CD9 antibody (clone MZ3), APC-conjugated anti-mouse CD63 antibody (clone NVG-2), or APC-conjugated rat IgG_2a_, k isotype control antibody (clone RTK2758) (all from BioLegend, San Diego, CA, USA) for 1 h at room temperature. After washing with exosome binding enhancer-containing washing buffer, the samples were analyzed using a FACS Aria Fusion (Beckton Dickinson, San Jose, CA, USA).

Particle sizes of EVs were measured using a Zetasizer Nano ZSP (Malvern Panalytical, Malvern, UK).

### 4.6. Effect of EVs on IL-5 Production from ILC2s 

As previously reported [24], ILC2s were sorted from the lung cells by flow cytometry. Briefly, all lung lobes were isolated 24 h after the 4th challenge under general anesthesia and minced in PBS. Cell suspensions were obtained by digesting all lung lobes using 870 units/mL of collagenase type I (Thermo Fisher Scientific, Waltham, MA, USA) at 37 °C for 1 h. Cells were treated with ACK lysis buffer to remove erythrocytes. The total number of leukocytes was counted using a hemocytometer after staining with trypan blue (Thermo Fisher Scientific, Waltham, MA, USA).

In accordance with a previous report (paper), ILC2s were detected and sorted as lineage^−^ CD45^+^ CD278^+^ CD90.2^+^ ST2^+^ cells. First, the leukocytes were treated with an anti-mouse CD16/32 (FcγRII/III) antibody to prevent non-specific binding of the subsequently used antibodies to the cells. After washing with 2% fetal bovine serum-containing PBS, the cells were stained with fluorescein isothiocyanate-conjugated anti-mouse lineage cocktail including anti-mouse CD3ε (clone 145-2C11), anti-mouse Ly-6G/Ly-6C (clone RB6-8C5), anti-mouse CD11b (clone M1/70), anti-mouse CD45R/B220 (clone RA3-6B2), anti-mouse TER-119/Erythroid cells (clone Ter-119), Pacific blue-conjugated anti-mouse CD45 antibody (clone 30-F11), phycoerythrin-conjugated anti-mouse CD278 antibody (clone 15F9), brilliant violet 510-conjugated anti-mouse CD90.2 (Thy-1.2) antibody (clone 53-2.1) (all from BioLegend, San Jose, CA, USA), and APC-conjugated anti-mouse ST2 antibody (clone RMST2-2, Thermo Fisher Scientific, Waltham, MA, USA) for 20 min at 4 °C. After washing with 2% fetal bovine serum-containing PBS, ILC2s were sorted as lineage^−^ CD45^+^ CD278^+^ CD90.2^+^ ST2^+^ cells by using a FACS Aria fusion. 

The sorted ILC2s (5 × 10^4^ cells/mL) were seeded in 96-well plates at 200 μL/well. Then, a mixture of IL-33 (BioLegend, San Jose, CA, USA, 1 μg/mL) and TSLP (R&D Systems, Minneapolis, MN, USA, 1 μg/mL) was added at 2 μL/well and incubated for 72 h at 37 °C. After incubation, the cell culture supernatants were collected, followed by determination of the concentration of IL-5 using an IL-5 mouse ELISA kit (Thermo Fisher Scientific, Waltham, MA, USA).

### 4.7. Statistical Analyses

One-way ANOVA was performed to compare multiple groups, followed by Dunnett’s multiple comparison test. The difference was detected as significant when the *p*-value was less than 0.05. These calculations were conducted using JMP Pro (Version 15.1.0, SAS Institute Japan, Tokyo, Japan).

## Data Availability

Not applicable.
